# Possible Association between Expression of Chemokine Receptor-2 (CCR2) and Amyotrophic Lateral Sclerosis (ALS) Patients of North India

**DOI:** 10.1371/journal.pone.0038382

**Published:** 2012-06-07

**Authors:** Pawan K. Gupta, Sudesh Prabhakar, Neel K. Sharma, Akshay Anand

**Affiliations:** Neuroscience Research Laboratory, Department of Neurology, Post Graduate Institute of Medical Education and Research (PGIMER), Chandigarh, India; Brigham and Women’s Hospital -, Harvard Medical School, United States of America

## Abstract

**Background and Objectives:**

We earlier reported elevated chemokine ligand-2 (CCL2) in Indian amyotrophic lateral sclerosis (ALS) patients. We now analysed chemokine receptor-2 (CCR2), the receptor of CCL2, in these ALS patients.

**Methods:**

Indian sporadic ALS patients (n = 50) were included on the basis of El Escorial criteria. Percentage (%) of CCR2 expressing peripheral blood mononuclear cells (PBMCs) was evaluated using Flow Cytometry. Real Time Polymerase Chain Reaction (PCR) was used to quantitate CCR2 mRNA expression in PBMCs. Normal controls (n = 40) were also included for comparison.

**Results:**

Flow Cytometry revealed significantly reduced CCR2 expressing PBMCs in the ALS patients. We also found a significant decline in number of CCR2 expressing PBMCs in limb onset ALS when compared to bulbar onset ALS. PBMCs from ALS patients showed substantial down-regulation of CCR2 mRNA. CCR2 mRNA expression was found to be decreased among limb ALS patients as compared to bulbar onset ALS. Further, the count of CCR2+ PBMCs and CCR2 mRNA transcript in PBMCs was significantly lower in severe and moderate ALS as compared to ALS patients with mild impairments.

**Conclusions:**

Downregulation of PBMCs CCR2 may indicate its etio-pathological relevance in ALS pathogenesis. Reduced PBMCs CCR2 may result in decreased infiltration of leukocytes at the site of degeneration as a compensatory response to ALS. CCR2 levels measurements in hematopoietic stem cells and estimation of comparative PBMCs count among ALS, disease controls and normal controls can unveil its direct neuroprotective role. However, the conclusions are restricted by the absence of neurological/non-neurological disease controls in the study.

## Introduction

Amyotrophic lateral sclerosis (ALS) is a fatal neurodegenerative disease characterized by typical participation of inflammatory cascade. Interaction of chemokine ligand-2 (CCL2), a small chemokine belongs to C-C subfamily with its receptor chemokine receptor-2 (CCR2) strongly regulate these inflammatory changes. CCL2/CCR2 pathway is known to drive circulating leucocytes and resident immune cells of brain, including microglial cells, towards the site of neurodegeneration. Studies have shown that CCL2 and CCR2 knock out transgenic mouse exhibit reduced infiltration of blood mononuclear, natural killer cells and dendritic cells at the site of inflammation and these mice are resistant to experimental autoimmune encephalomyelitis (EAE) [1], [2]. Furthermore, elevated CCL2 levels in biofluids from ALS patients have been reported earlier [3]–[13]. Contrary, reduced CCR2 expression in peripheral blood monocytes of ALS patients has also been observed [7], [14] and could be argued as conflicting with postulated role of CCR2 in inflammation in ALS pathogenesis. Therefore more studies in other populations with varying clinical phenotype are imperative to uncover the role of interplay of these molecules in the ALS disease.

Whether CCL2/CCR2 alteration is neurotoxic or provides neuroprotection at a given stage of ALS disease remains unclear as CCL2/CCR2 pathway is also reported to impart neuroprotection besides mediating inflammation [15], [16]. For instance, it has been demonstrated that CCL2 rescues fetal neurons and astrocytes in a mixed culture from N-methyl-D-aspartate (NMDA) induced apoptosis by reducing glutamate and NMDA receptor-1 (NMDAR1) [15]. Additionally, CCL2/CCR2 has also been reported to prevent HIV-tat induced damage of rat midbrain neurons [16].

We recently reported higher CCL2 in bio-fluids from Indian ALS patients and postulated that this may contribute towards extended survival reported in these patients [12], [13]. A major study from India reported significantly longer survival duration among ALS patients when compared to Western ALS populations [17]. In this study, we present an indirect evidence of reduced mRNA and protein CCR2 levels in peripheral blood mononuclear cells (PBMCs) of Indian ALS patients suggesting its etio-pathological association with ALS.

## Materials and Methods

### Ethics Statement

All subjects were included in the study after obtaining written informed consent as outlined in the research protocol. Ethical approval for the study was obtained by institute ethical committee, Post Graduate Institute of Medical Education and Research (PGIMER), Chandigarh, India–160012 (No. 7055-PG-1Tg-05/4348-50).

**Table 1:** Characteristics of subjects.

Subjects	†Age (y)	M/F (n)	Age of onset (y)	‡,†Disease duration (mo)	El Escorial criteria at the time of sample collection	†Total serum protein (g/l)
Total ALS	47.4±12.4	38/12	46.2±12.8	19.0±12.7	25 Definite, 15 Probable, 10 Possible	48.2±26.7
Limb onset ALS	47.0±13.3	33/9	45.5±13.8	19.5±14.0	20 Definite, 15 Probable, 7 Possible	47.6±26.7
Bulbar onset ALS	49.7±7.5	5/3	48.3±7.3	15.7±6.3	5 Definite, 3 Possible	51.6±28.4
Normal controls	46.0±10.9	30/10				49.9±28.2

Clinical details of subjects. ALS, amyotrophic lateral sclerosis; F, female; g, grams; l, litre; n, Number; M, male; mo, months; y, years. Age, age of onset, duration of disease and total serum protein are indicated as mean ± standard deviation (SD).

‡ Duration of disease is the interval between appearance of first symptom of ALS and collection of sample.

† *One-way analysis of variance* (ANOVA) followed by Fisher’s least significant difference (LSD) *post hoc* analysis showed that mean age, mean disease duration and mean total serum protein did not differ significantly among the given conditions (p>0.05). ALS subjects were asked to provide all clinical details at the age of onset of disease.

**Figure 1:**
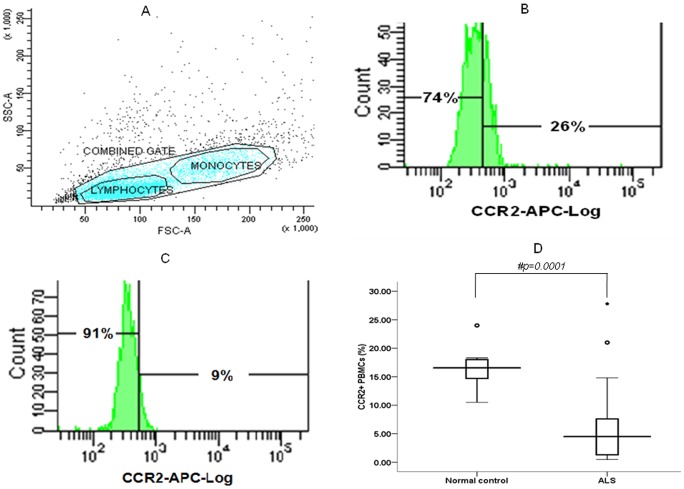
Percentage (%) of CCR2+PBMCs in ALS patients and normal control subjects as measured by Flow Cytometry. (A) Dot plot showing side and forward scatter analysis of purified unlabeled PBMCs (large combined gate) from a normal control. PBMCs consists of two distinct populations namely lymphocytes and monocytes. Approximate lymphocytes and monocytes populations are indicated as smaller gates. Events outside the PBMCs gate represent cell debris and granulocytes. Same gating has been used for PBMCs from each ALS and normal control sample. ∼10,000 events have been acquired in each experiment. x*-*axis represents population cell size in forward scatter (FSC) and y-axis denotes population cell granularity in side scatter (SSC). (B,C) Single parameter representative histogram of flow cytometric expression pattern of CCR2 on gated PBMCs is showing decreased number of CCR2 expressing PBMCs in ALS (9%; C) as compared to normal control (26%; B). Number of cells is represented along y-axis and blue APC fluorescence along x-axis. Appropriate unlabeled PBMCs were used to set marker in histogram and measure background fluorescence. (D) Box plot compares CCR2 expressing PBMCs between ALS and normal subjects. Boxes include values from first quartile (25th percentile) to third quartile (75th percentile). Lower and upper error bar refers to 10th and 90th percentile respectively. The thick horizontal line in the box represents median for each dataset. Outliers and extreme values are shown in circles and asterisk respectively. Nonparametric *Mann Whitney U* test indicates significant difference betweesn the given conditions (p<0.05). ALS, amyotrophic lateral sclerosis; APC, allophycocyanin; CCR2, chemokine receptor 2; PBMCs, peripheral blood mononuclear cells.

**Figure 2:**
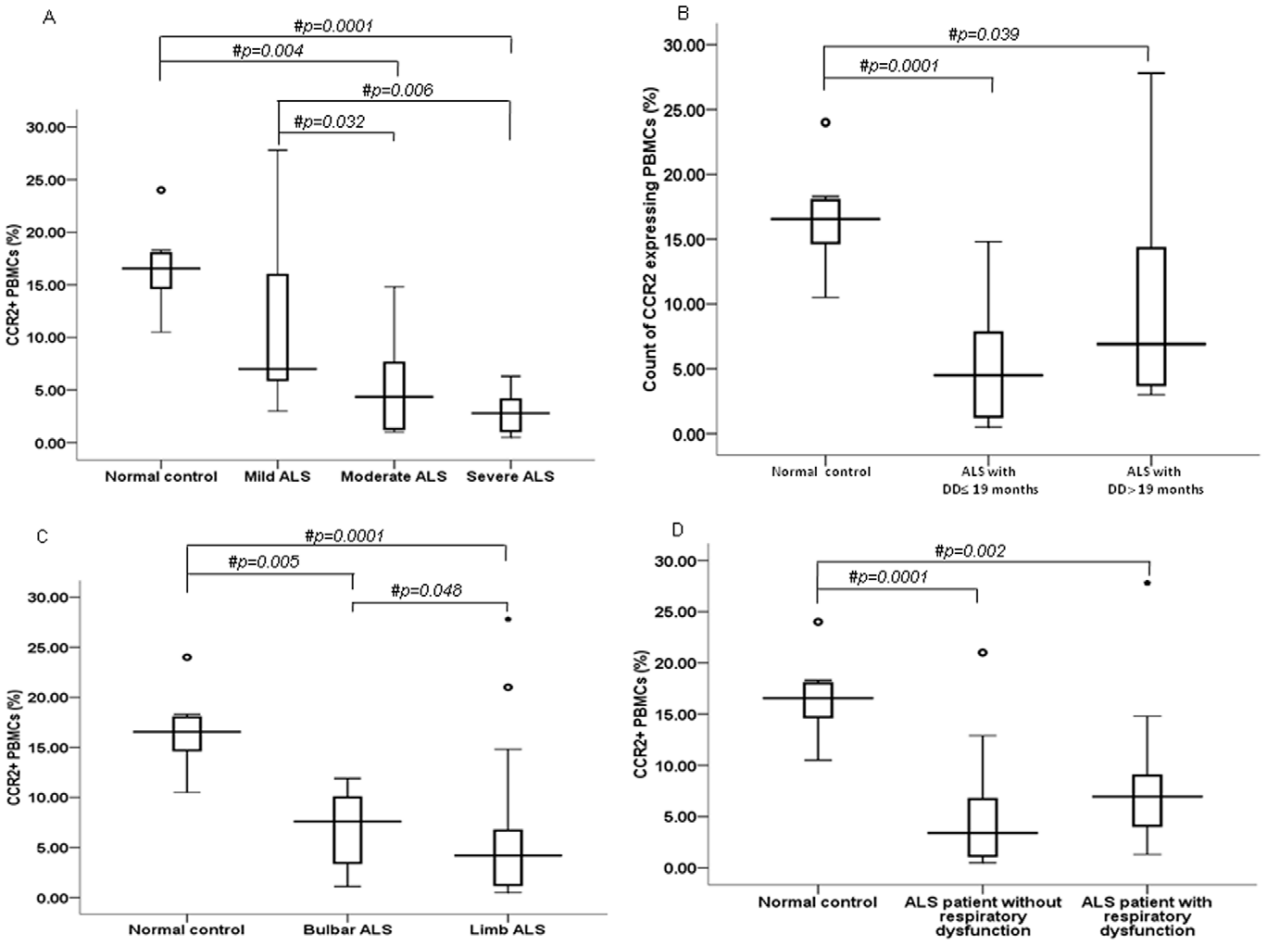
CCR2+ PBMCs in ALS patients with varying clinical characteristics. (A) Percentage (%) of PBMCs expressing CCR2 protein in ALS patients with mild, moderate and severe neurological impairments as indicated by ALSFRS-R. (B) Count (%) of CCR2+ PBMCs in ALS subjects with disease duration (DD) ≤19 months and DD>19 months. (C) Percentage (%) of CCR2 expressing PBMCs in bulbar and limb onset ALS patients. (D) CCR2+ PBMCs in ALS patients with respiratory dysfunction. In each box plot (A–D), boxes include values from first quartile (25th percentile) to third quartile (75th percentile). Lower and upper error bar refers to 10th and 90th percentile respectively. The thick horizontal line in the box represents median for each dataset. Data was collected using Flow Cytometry. A non-parametric *Kruskal-Wallis H* test followed by *Mann Whitney U* test was used to analyze the data. # indicates significant difference among the groups (p<0.05). Outliers and extreme values are shown in circles and asterisk respectively. ALS, amyotrophic lateral sclerosis; ALSFRS-R, ALS functional rating score-revised; CCR2, chemokine receptor 2; DD, disease duration; PBMCs, peripheral blood mononuclear cells.

**Figure 3:**
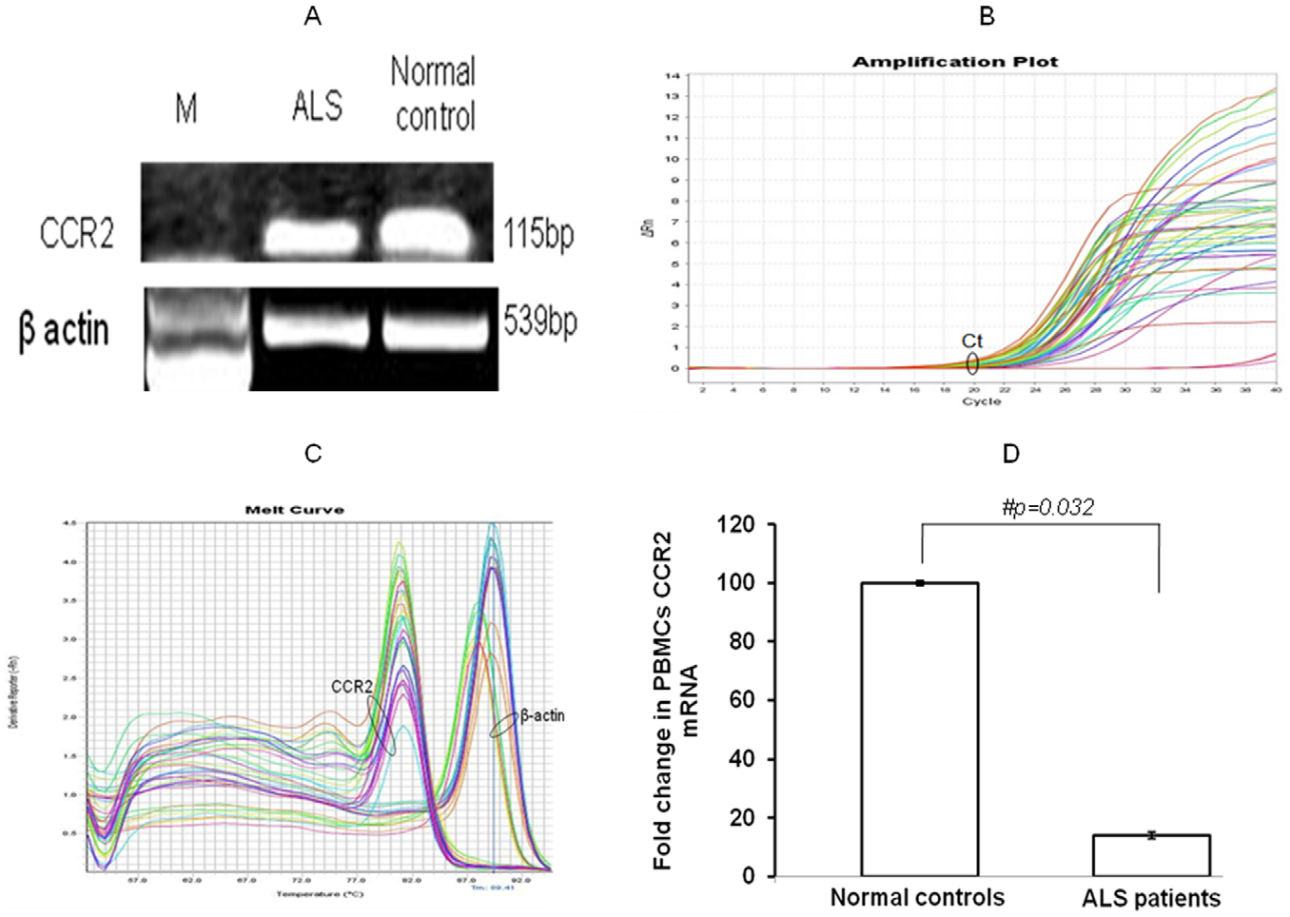
Real Time PCR analysis of relative mRNA expression of CCR2 in PBMCs of subjects. (A) Agarose gel electrophoresis of Real Time PCR products of target gene CCR2 and endogenous control β-actin. Reactions were performed with mRNA isolated form PBMCs of ALS patient and normal control. (B) Representative amplification curves depicting increase in fluorescence of CCR2 and β-actin from cDNA of same sample. No increment in fluorescence of negative controls was observed. The x-axis indicates cycle number and the y-axis shows intensity of relative fluorescence in linear scale. Ct is threshold cycle where normalized fluorescent signal of SYBR green intersect with threshold line. (C) Melt curve profile of Real Time PCR products of CCR2 and β actin clearly indicates the absence of any non specific amplification. The x-axis indicates negative derivative of change in amount of fluorescence per unit change in temperature (dF/dT) and the y-axis represents temperature in Celsius (°C). (D) Bar diagram showing fold change in CCR2 mRNA expression PBMCs from ALS and normal subjects. Values are plotted as mean ± SE (Standard error). Data was analyzed by unpaired, independent 2-tailed *student t* test with equal variance. # indicates significant difference among the groups (p<0.05). Expression of CCR2 were normalized to expression of endogenous control β-actin. ALS, amyotrophic lateral sclerosis; CCR2, chemokine receptor 2; PBMCs, peripheral blood mononuclear cells.

**Figure 4:**
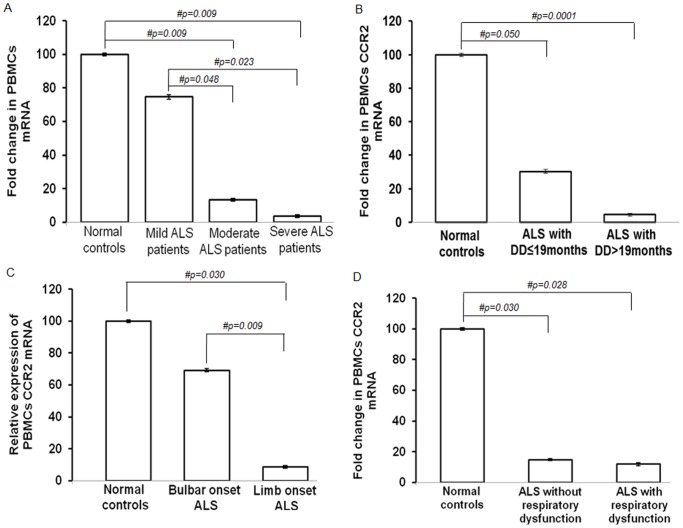
CCR2 mRNA in PBMCs of ALS patients with different clinical states. (A) Relative mRNA expression of CCR2 in PBMCs from ALS patients with mild, moderate and severe neurological impairments as indicated by ALSFRS-R. (B) Fold change in expression of PBMCs CCR2 mRNA in ALS subjects with disease duration (DD) ≤19 months and DD>19 months. (C) Relative CCR2 mRNA expression in PBMCs of bulbar and limb onset ALS patients. (D) Comparison of PBMCs CCR2 transcripts in ALS patients with respiratory dysfunction. In each bar diagram (A–D), values are plotted as mean ± SE (standard error). Data was collected using Real Time PCR and analyzed by *one-way analysis of variance* (ANOVA) followed by Fisher’s least significant difference (LSD) *post hoc* analysis. # indicates significant difference among the groups (p<0.05). Expression of CCR2 was normalized to expression of endogenous control β-actin. ALS, amyotrophic lateral sclerosis; ALSFRS-R, ALS functional rating score-revised; CCR2, chemokine receptor 2; DD, disease duration; PBMCs, peripheral blood mononuclear cells.

### Subjects

Fifty patients, born in North India and diagnosed with ALS by El Escorial criteria at Neurology outpatient, PGIMER, Chandigarh, India were recruited in the study. ALS patients with history of diabetic neuropathy, glaucoma, pre-eclampsia, stroke and those receiving Riluzole, anti inflammatory drugs, antioxidants or other treatment were excluded from the study. The ALS functional rating score-revised (ALSFRS-R) was measured to evaluate severity of disease and overall functional status of patients. This revealed 11 patients which presented with respiratory symptoms such as orthopnea, dyspnea and other respiratory insufficiencies even though none of the patients were on respiratory support [18]. At the time of blood collection, 15 ALS patients presented with mild neurological impairment [ALSFRS-R_range_ = 36–45; ALSFRS-R_mean_ = 40±0.5(SE)], 30 ALS patients with moderate impairment [ALSFRS-R_range_ = 24–36; ALSFRS-R_mean_ = 32.5±0.4(SE)] while 5 ALS patients with severe clinical phenotype [ALSFRS-R_range_ = 16–24; ALSFRS-R_mean_ = 18.5±1.5(SE)] as indicated by ALSFRS-R criteria. The ALS patients had an overall mean ALSFRS-R score of 34.4±0.8(SE) with a range of 16 to 45. Disease duration (interval between appearance of first ALS symptom and sample collection) for patients with mild, moderate and severe impairments is reported to be 16.6±11.6(SD), 18.4±11.9(SD) and 28.8±23.0(SD) months respectively. Of 8 bulbar onset ALS patients, 3 patients exhibited severe neurological impairments and remaining 5 were presented with moderate impairments. Of 42 limb onset ALS cases, 2 patients were severe, 15 cases were presented with mild deficit and 25 were presented with moderate neurological deficit. Based on disease duration, ALS patients were divided in two groups, disease duration≤19 months (n = 34) and disease duration>19 months (n = 16). The mean disease duration of ALS patients was 19.0±12.7(SD) months and therefore set as cutoff. The reference group for comparisons consisted of 40 genetically unrelated; sex and age matched normal controls without any apparent health problems such as hypertension, diabetes, heart disease etc. Since there were no neurological deficits upon examination, the ALSFRS-R score of each normal control individual was considered as 48. No neurological/nonneurological disease controls were included in the study. The clinical details of subjects which are published earlier [12], have also been reproduced here in Table 1.

### PBMCs Isolation

PBMCs were isolated as per Histopaque-1077 (Sigma, USA) instruction sheet provided by the vendor. Briefly, 6.0 ml blood was collected from each subject and layered on equal volume of Histopaque-1077 followed by centrifugation at 1800 rpm for 30.0 mins at room temperature. PBMCs were collected from plasma/Histopaque-1077 interface. Aliquots of PBMCs were stored at −80°C in *RNA later* (Sigma, USA) until used for total RNA extraction. Some aliquots of PBMCs was stored in 90% fetal bovine serum (FBS, HiMedia, India) +10% dimethyl sulphoxide (DMSO, Sigma, USA) and kept at −80°C until flow cytometry was done.

### RNA Extraction

Total RNA was extracted using RNAeasy columns (Qiagen, USA). RNA was quantitated by taking absorbance at 260.0 nm. About 5000.0 ng total RNA was used to synthesize cDNA according to RevertAid™ first strand cDNA kit (Fermentas, USA).

### Real Time Polymerase Chain Reaction (PCR)

Real Time PCR was used to quantitate expression of CCR2 mRNA in PBMCs and was performed in the 48 wells version of Step One™ (Applied Biosystems Inc., USA) using CCR2 specific forward (5′-AGT TCA GAA GGT ATC TCT CGG TC-3′) and reverse primer (5′-GGC GTG TTT GTT GAA GTC ACT-3′) sequences available at primer bank (http://pga.mgh.harvard.edu/cgi-bin/primerbank). PCR reactions were carried out in duplicates using SYBR green real time PCR kit (Invitrogen, USA) according to manufacturer’s recommendations. The cycling conditions consisted of initial denaturation step for 10.0 mins at 95°C followed by 40 cycles of denaturation at 95°C for 1.0 min, annealing at 52°C for 1.0 min and extension at 72°C for 1.0 min. Relative expression was analyzed using 2^−ΔΔCt^ or comparative Ct (threshold cycle) method after normalization with β-actin [4] and fluorescence data were obtained at annealing step.

### Flow Cytometry

Flow Cytometry was used to study CCR2 protein levels on PBMCs surface. ∼ 3×10^5^ PBMCs were blocked with Fc blocker (1.0 µg, purified human IgG, R&D Systems Inc., USA) +0.1% sodium azide (Sigma, Germany) +1X Ca^2+^ and Mg^2+^ free phosphate buffer saline (PBS, HiMEDIA, India), for 15.0 mins at room temperature. Cell suspension was then stained with anti-human CCR2 primary antibody labeled with allophycocyanin (0.1 µg, R&D Systems Inc., USA) +0.5% bovine serum albumin (BSA, Sigma, Germany) +0.1% sodium azide +1X PBS for 45.0 mins on ice in dark followed by two washings with 1X PBS at 5,000 rpm for 5.0 mins at 4°C. Finally, the cells were reconstituted in 250.0 µl of 1X PBS and analyzed in FACSCANTO (BD Biosciences, USA) flow cytometer using FACS DIVA software with in 1 hr. Approximately 10,000 viable PBMCs were gated based on their forward and side scatter profile, and acquired in each run. PBMCs gate was set to include both lymphocytes and monocytes where maximum CCR2 fluorescence was observed. Events outside the PBMCs gate represent cell debris and any contaminated granulocytes while separating PBMCs from whole blood. Same gating was used between the experiments. Since purified population of PBMCs was used during flow cytometry experiments, no additional surface marker labeling was done to identify such populations. Background signal was measured for each sample by acquiring unlabeled PBMCs as negative controls and normalized to the signal obtained from anit-hCCR2 labeled PBMCs.

### Statistical Analysis

For skewed data and multiple comparisons, a non-parametric *Kruskal-Wallis H* test followed by *Mann-Whitney U* test was applied. For skewed data and comparison of two groups, a nonparametric *Mann-Whitney U* was used to score the level of significance.

A parametric *one-way analysis of variance* (ANOVA) followed by Fisher’s least significant difference (LSD) *post hoc* test was applied to compare multiple groups with normal distribution. However, if data was normally distributed with two groups to compare, unpaired, independent 2-tailed student *t* test with equal or unequal variance (Welch’s correction) was applied. Quantile-quantile (Q-Q) plot was used to understand whether data is normally distributed or skewed. Skewed and normally distributed data is shown as median (10^th^ percentile −90^th^ percentile) and mean ± standard error (SE) respectively. The *p-*value was considered significant at ≤0.05. All statistical analysis was performed by statistical package and service solution (SPSS) 16 software.

## Results

Flow Cytometry analysis of PBMCs of ALS and normal controls indicates a significant decrease in proportion of CCR2 expressing PBMCs in ALS patients than normal controls (Figure 1A–1D; p = 0.0001). CCR2 expressing PBMCs were found to be lower in severe ALS than ALS patients with mild and moderate neurological impairment (Figure 2A; p = 0.006 and p = 0.032 respectively). No such difference was observed in ALS patients with varying duration (Figure 2B; p>0.05). Reduced CCR2+ PBMCs was observed in bulbar and limb variants of ALS as compared to control group (Figure 2C; p = 0.005 and p = 0.0001 respectively). Moreover, CCR2+ PBMCs were found to be lower in limb onset ALS than bulbar onset ALS patients (Figure 2C; p = 0.048). In order to examine any possible association with hypoxia the CCR2 levels were also analysed in ALS patients with respiratory dysfunction and those without respiratory dysfunction, however, no difference in CCR2 was been observed between these ALS groups (Figure 2D; p>0.05).

Real Time PCR analysis also indicated a 7.1-fold down-regulation of CCR2 mRNA expression in PBMCs of ALS patients as compared to normal subjects (Figure 3A–3D; p = 0.032). A 27-fold and 20-fold reduction was observed in severe ALS as compared to normal and mild ALS respectively (Figure 4A; p = 0.009 and p = 0.023 respectively), however, mRNA levels were comparable across moderate and severe ALS patients (Figure 4A; p>0.05). In addition, there was no significant reduction of CCR2 mRNA in PBMCs from ALS patients with disease duration >19 months in comparison to ALS patients with ≤19 months (Figure 4B; p>0.05). There was a significant decrease of 8.0-fold in PBMCs CCR2 mRNA in limb onset ALS when compared with bulbar onset ALS patients (Figure 4C; p = 0.009). PBMCs CCR2 transcript expression was comparable between ALS patients with respiratory dysfunction and without respiratory problems (Figure 4D; p>0.05).

## Discussion

It has earlier been established that chronic activation of PBMCs via CCL2/CCR2 signaling pathway mediates inflammation in many neurological disorders. It has been observed that infiltration of PBMCs in the central nervous system (CNS) of mouse model of EAE is mediated by CCR2 [19], [20]. A profound reduction in infiltration of leukocytes has been reported around denervated hippocampus following axonal injury in CCR2 deficient mice [21]. Mahad et al., showed that the *in vitro* model of blood-brain barrier (BBB) was selectively permeable for migrating CCR2+ lymphocytes and monocytes, and suggest the pathological importance of infiltrated CCR2 expressing PBMCs in multiple sclerosis (MS) [22]. The concept is furthered by the observation that the ablation of CCR2 in HexB−/− mouse model of Sandhoff disease results in reduced PBMCs infiltration in the brain parenchyma and ameliorates the clinical progression of the disease by reducing neuroinflammation, and hence links reduction in CCR2+ PBMCs with neuroprotection [23]. Increased production of inflammatory chemokines has recently been reported in PBMCs of Alzheimer’s disease (AD) patients [24]. With this background, we measured the levels of CCR2 in PBMCs of Indian ALS patients as these patients exhibit substantially extended survival duration of ∼115 months after onset of disease [17].

The reduced systemic expression of CCR2 in PBMCs of these ALS patients reported here suggests its etio-pathological relevance to ALS pathogenesis. Earlier elevated levels of abnormally activated and differentiated monocytes/macrophages in sporadic ALS patients [25] were found to be associated with down regulation of CCR2 on circulating monocytes [7]. Furthermore, a significant reduction of CD14+ and CCR2 expressing monocytes in ALS patients, particularly with less severe form of disease, has been suggested to drive the recruitment of activated monocytes CNS in the early stages of the disorder [14].

We propose that the decrease in PBMCs CCR2 and previously reported elevated CCL2 in our ALS patients [12], [13] may indicate an activation of a negative feedback regulation serving to alleviate the inflammation caused by extravasation of activated monocytes/lymphocytes at the site of CNS injury and denervated neuromuscular junction. Significantly reduced CCR2 levels in moderate and severe ALS patients and not in mild variants show that CCR2 may not be causally associated with primary motor neuron degeneration and its pathophysiological involvement could be secondary to neurodegeneration. However, our finding of unaltered CCR2 levels across ALS patients with varying disease duration is explained by absence of information about disease progression rate and actual survival duration after onset of disease. Therefore, at this time, direct association of reduced CCR2 with extended survival duration of these ALS patients awaits further analysis through multi ethnic and multi cultural studies.

Reduced CCR2+ PBMCs in peripheral blood at the time of sample collection may raise the possibility of their migration and extravasation in CNS through damaged blood-brain barrier (BBB) and blood-spinal cord barrier (BSCB) in ALS pathogenesis [26], [27], [28], [29], however, anatomical and histopathological analysis of BBB and BSCB was not conducted. Our report, therefore, suggests the need of future autopsy studies where brain and spinal cord tissue from Indian ALS patients can be analysed for presence of CCR2+ PBMCs. The massive infiltration of immature blood dendritic cells, CD4+ and CD8+ T-lymphocytes in spinal cord parenchyma has earlier been observed in Western ALS cases [4] and in superoxide dismutase 1 (SOD1) mutated transgenic ALS mouse model [30].

The unaltered levels of total serum protein in the ALS patients studied (Table 1) suggest that these findings are specific to CCR2+ PBMCs. However the expression of CCR2+ immune cells other than PBMCs including natural killer cells, dendritic cells and macrophages in these patients were not separately performed. Significantly elevated PBMCs CCR2 in bulbar ALS versus limb variant appears to be in contrast to the theory developed in the paper. For instance, the overall functional deterioration was found to be higher in bulbar [Mean ALSFRS-R = 28.7±3.2(SE)] when compared to the limb ALS [Mean ALSFRS-R = 35.4±0.8(SE)]. We speculate that higher levels of PBMCs CCR2 reported in bulbar variants may result from reduced disease duration and severity of these cases. Hence, future longitudinal studies for estimation of PBMCs CCR2 in higher number of bulbar Indian ALS patients and its possible association with clinical progression of the ALS disease may provide useful information about role of CCR2 in ALS. Since lower PBMCs CCR2 may facilitate neuroprotection, at the moment, lower CCR2 levels among limb ALS than bulbar ALS patients may account for relatively slower progression of disease and longer survival duration in limb ALS cases [17], [31]. These findings are consistent with existing literature where limb Indian ALS patients were reported to exhibit significantly higher median survival duration [177.9±3.2(SE)] after disease onset as compared to bulbar group [55.9±2.9(SE)] [17].

We further propose that the downregulation of CCR2 mRNA in ALS subjects may indicate the underlying genetic/epigenetic abnormalities in regulatory elements of CCR2 gene including its post transcriptional deregulation necessitating detailed analysis of CCR2 domain in ALS population. CCR2 reduction in response to environmental cues can also not be ruled out in present ALS patients. Even though respiratory dysfunction did not impact CCR2 levels in the present study (Figure 2D & 4D), role of respiratory impairment should be addressed in larger ALS cohort with respiratory complications as downregulation of monocytic CCR2 under hypoxia has earlier been observed in *in vitro* conditions [32].

Because of lack of neurological disease controls having overlapping or distinct clinical symptoms with ALS, the differences observed in CCR2 levels in the study may reflect the molecular change relevant to diseases of CNS in general, as opposed to ALS disease in specific. Therefore, the absence of such control group is an important caveat of the study and further investigations should focus on the use of neurological controls in the analysis.

In conclusion, although causal association of reduced CCR2 with increased survival in Indian ALS patients remains speculative, the present findings may suggest an etio-pathological and possible immunomodulatory importance of PBMCs CCR2 in pathogenesis of ALS. Whether the blocking or reducing glial and leukocyte CCR2 (by intracerebral and/or systemic injection of its antagonists or synthetic siRNA against CCR2 mRNA) will lead to any therapeutic efficacy in ALS will be determined by further preclinical studies.
